# Variations in osmotic adjustment and water relations of *Sphaerophysa kotschyana*: Glycine betaine, proline and choline accumulation in response to salinity

**DOI:** 10.1186/1999-3110-55-6

**Published:** 2014-01-17

**Authors:** Evren Yildiztugay, Ceyda Ozfidan-Konakci, Mustafa Kucukoduk, Yagmur Duran

**Affiliations:** 1 Department of Biology, Selcuk University, Faculty of Science, Selcuklu, 42031 Konya, Turkey; 2 Department of Molecular Biology and Genetics, Faculty of Science, Necmettin Erbakan University, Meram, 42090 Konya, Turkey

**Keywords:** Glycine betaine, Osmotic adjustment, Proline, *Sphaerophysa kotschyana*, Water relations

## Abstract

**Background:**

*Sphaerophysa kotschyana* Boiss. is naturally distributed in overly salty regions. The key to the completion of the life cycles of *S. kotschyana* in harsh saline soils may be hidden in changes of its osmo-protectants, but there is currently no information about the interaction between osmotic adjustment and water relations in adaptation to saline conditions. The aim of this article was to determine growth, relative growth rate (RGR), relative water content (RWC), osmotic potential (Ψ_Π_), photosynthetic efficiency (F_v_/F_m_), thiobarbituric acid-reactive substances (TBARS) and osmo-protectant contents [proline (Pro), choline (Cho) and glycine betaine (GB)] in *S. kotschyana* leaves and roots exposed to 0, 150 or 300 mM NaCl for 7 and 14 d (days).

**Results:**

The results clearly showed that the reductions in growth, RWC, F_v_/F_m_, RGR and Ψ_Π_ were more pronounced at 300 mM, especially after 14 d. In the same group, the highest increase in TBARS was recorded in roots (126%) and leaves (31%). The induction at 150 mM was not as high. Therefore, roots appear to be the most vulnerable part of this plant. Moreover, *S. kotschyana* was able to withstand short-term low salinity.

**Conclusions:**

The osmo-protectant accumulation in *S. kotschyana* as a salinity acclimation or adaptation was sufficient for toleration of low salt concentration (150 mM). In contrast, the plants exposed to the highest NaCl concentration (300 mM) were not able to maintain the ability to prevent water loss because of further decrease in root/shoot ratio of fresh weight (FW) and dry weight (DW), RWC and RGR.

**Electronic supplementary material:**

The online version of this article (doi:10.1186/1999-3110-55-6) contains supplementary material, which is available to authorized users.

## Background

About one-fifth of irrigated world agricultural lands are adversely affected by salinity, leading to induction of a wide range of perturbations at cellular and whole-plant levels (Belkheiri and Mulas, 2011). Salt stress may provoke (i) osmotic or water-deficit effect which cause reduction of water and nutrient uptake and (ii) ion-excess effect resulting from altered K^+^/Na^+^ ratios and/or accumulation of toxic levels of Na^+^ and Cl^¯^. Salt stress-induced oxidative stress results from excess reactive oxygen species (ROS) formation (Munns and Tester, 2008) which damages membrane lipids, proteins and nucleic acids (Mittler, 2002). In the cellular adaptive responses of salt-tolerant plants, a major factor of salt tolerance mechanisms is not only acceleration of ROS scavenging systems (Chinnusamy et al., 2005), but also the ability of plant cells to adjust osmotically and to accumulate organic solutes known as compatible solutes. Compatible solutes referred to as osmo-protectants are thought to provide (i) positive effects on cellular components and membrane integrity (ii) maintenance of subcellular structure and cellular turgor and (iii) increase in the osmotic potential of the cell in plants subjected to stress conditions (Sakamoto and Murata, 2002; Ashraf and Foolad, 2007). These osmo-protectant solutes include proline (Pro) and, quaternary ammonium compounds such as glycine betaine (GB) (Belkheiri and Mulas, 2011).

Pro is believed to play adaptive roles in plant stress tolerance. Pro has been proposed to act as (i) a compatible osmolyte, (ii) a molecular chaperone stabilizing the structure of proteins, (iii) a mechanism to store carbon and nitrogen, (iv) to balance cell redox status and (v) a part of stress signal influencing adaptive responses (Ashraf and Foolad, 2007; Verbruggen and Hermans 2008). Among the compatible solutes, GB is a small organic metabolite abundant mainly in the chloroplast, that stabilizes the activities of enzymes/protein complexes and maintains the integrity of membranes against the damaging effects of various environmental stresses (Sakamoto and Murata, 2002). The relationship between GB content and increasing in salinity, however, is controversial. For example, GB accumulates in response to stress in many plants, including *Nicotiana tabacum* and *Atriplex halimus* (Banu et al., 2009; Belkheiri and Mulas, 2011). In contrast, contradictory data has been obtained in *Trifolium alexandrinum* and *Salicornia dolichostachya* by different researchers (Varshney et al., 1988; Katschnig et al., 2013). Therefore, GB-induced defensive responses under salt stress have still been a matter of some confusion and debate. On the other hand, choline (Cho), was synthesized by GB in the chloroplast, has a vital role as the precursor for phosphatidyl choline, a dominant constituent of membrane phospholipids in eukaryotes (Sakamoto and Murata, 2002). In the literature, accumulation of these compatible solutes has been extensively investigated under stress conditions, but not interaction between these osmo-protectants. Hence, the interplays among Pro, Cho and GB accumulations need to be assessed, especially in halophytes having natural defense systems against salinity.

The Salt Lake (Tuz Gölü, Turkey), the habitat of *Sphaerophysa kotschyana* (Fabaceae)*,* features a unique ecosystem with its natural attractive environments and habitats for biota. Although salinity level changes with seasonal fluctuations, this lake water is extremely saline with a salt content of 32%. The lake bottom is covered with a 1 to 30 cm thick salt layer, which has given rise to a local salt industry providing 55% of all Turkish salt (Dengiz and Baskan, 2009). *S. kotschyana* is naturally distributed in overly salty regions and is well equipped to survive and complete their life cycles in saline soils (Duran et al., 2010). It has been documented in previous studies that halophytic species have developed protective or compensatory mechanisms to resist salt stress, but there is no information about tolerance and/or avoidance defined by osmotic adjustment and tighter control of water relations in the *S. kotschyana* under salinity. Interestingly, how *S. kotschyana* can survive to the harsh salt conditions is not well understood. The answer to this question may be hidden in the change of its osmo-protectants under salt stress, as well as in antioxidant tolerance mechanisms. Nevertheless, in roots and leaves of *S. kotschyana*, no details are known about osmotic adjustment and water relations in tolerance processes in relation to changes of these osmo-protectants.

Rather than examining the most commonly studied osmolytes individually, we focused on the unifying features associated with the accumulation of Pro, Cho and GB. Also, the present work was conducted in order to assess to defense strategies produced by leaves and roots of *S. kotschyana* against salt stress. With this aim, the study was undertaken to comparatively to investigate the role of the accumulation of osmo-protectants such as Pro, Cho and GB on enhancing salt tolerance. Furthermore, these parameters were correlated with some physiological and biochemical parameters including growth, relative growth rate (RGR), leaf relative water content (RWC), osmotic potential (Ψ_Π_), chlorophyll fluorescence (F_v_/F_m_) and thiobarbituric acid-reactive substances (TBARS) concentration in leaves and roots of *S. kotschyana* exposed to 0, 150 and 300 mM NaCl for 7 and 14 d.

## Methods

### Plant material and experimental design

*Sphaerophysa kotschyana* Boiss. plant specimens and seeds were collected in July 2010 from around Yavsan Saltworks (Salt Lake), Konya (lat. 42°90′336“ long. 36°51′649” at an altitude of 910 m), Turkey. Taxonomic identification of the plant material was confirmed. The voucher specimen has been deposited at the Herbarium of the Department of Biology, Selcuk University, Konya, Turkey (Voucher No: EY2150). After seeds collection, immature seeds and those attacked by insects were removed and the healthy seeds were stored at 4°C.

*Sphaerophysa kotschyana* seeds were sterilized with 5% sodium hypochlorite for 10 min and were washed thoroughly with deionized water. Then, seeds were sown into the pots filled with perlite and were grown under controlled conditions (light/dark regime of 16/8 hours (h) at 23°C, 70% relative humidity and 350 μmol m^-2^ s^-1^ photosynthetic photon flux density). For determination of the range of salt concentration applied to *S. kotschyana*, 0 (control), 150, 300 and 400 mM NaCl was used in the pre-treatment experiment. The data obtained from it suggested that 150 and 300 mM were chosen for stress-induced oxidative damage and the plants subjected to 400 mM NaCl greatly damaged and very few of them could survive against stress treatment. Seedlings were grown for 50 d in a full-strength Hoagland’s solution (Hoagland and Arnon, 1950). On day 50 of normal growth (applied to seedling with five-leaf), a stress treatment was initiated by giving Hoagland’s solution containing 0, 150 and 300 mM NaCl. Plants were harvested on the 7 and 14 d of salt treatment and then stored at -80°C until further analyses.

### Growth and osmo-protectant accumulation of *S. kotschyana* under salinity

Six random plants for each group were used for measuring changes on the growth parameter. They were separated to shoot and root fractions on 7 and 14 d of stress and measured with the ruler for length. Shoot and root fresh weights (FW) of seedlings were weighed. After the samples were dried at 70°C for 72 h, dry weights (DW) were measured to calculate the relative growth rate (RGR). RGR values were calculated according to the following formula by Hunt et al. (2002):

$$\mathrm{RGR}=\left[\ln\left(DW_{2}\right)–\ln\left(DW_{1}\right)\right]/\left(t_{2}–t_{1}\right)$$

where DW_1_ = dry weight (g) at t_1_; DW_2_ = dry weight (g) at t_2_, t_1_; initial harvest and t_2_;final harvest.

After the stress exposure period, six leaves were weighted and their FW was recorded. The samples were kept in ultrapure water for 8 h and then the turgid leaves (TW) were measured again. After oven drying at 75°C for 72 h, DW was reported. RWC was calculated by the formula given by Smart and Bingham (1974):

$$\mathrm{RWC}\;\left(\%\right)=\left[\left(\mathrm{FW}-\mathrm{DW}\right)/\left(\mathrm{TW}-\mathrm{DW}\right)\right]\times 100$$

After harvest on 7 and 14 d of stress treatment, chlorophyll fluorescence parameters (PSII maximum efficiency, F_v_/F_m_) of leaves were measured by Plant Efficiency Analyze of Hansatech, UK. This parameter provided an estimate of the maximum photochemical efficiency of the photosystem II (PSII).

Samples were homogenized by a glass rod. After centrifugation (12000 × g) for 10 min, the extraction was directly used for Ψ_Π_ determination. Ψ_Π_ was measured by Vapro Vapor pressure Osmometer 5600. The data was collected from six samples per replicate. Ψ_Π_ was converted to MPa according to Santa-Cruz et al. (2002) by multiplying coefficient of 2.408 × 10^-3^.

Pro content was measured according to Bates et al. (1973). The samples were homogenized in 3% sulphosalicylic acid and homogenate was filtered through filter paper. After addition of acid ninhydrin and glacial acetic acid, the mixture was heated at 100°C. The mixture was extracted with toluene and the absorbance of the toluene fraction aspired from liquid phase was measured at 520 nm.

Cho level was determined according to Grieve and Grattan (1983). The dry sample material was suspended in 1 ml of distilled water and then was centrifuged at 5000 × g for 5 min. The supernatants were diluted 1:1 with 0.2 M potassium phosphate buffer (pH 6.8). After cooling the mixtures, 20 μl of cold KI-I_2_ reagent was added. The samples were stored at 0-4°C for 16 h and then centrifuged at 10000 × g for 15 min. Supernatant was removed and periodide crystals dissolved in 900 μl of 1,2-dichloroethane. After vortexing and incubating for 2–2.5 h at room temperature, the absorbance was recorded at 365 nm. GB content was performed according to Grieve and Grattan (1983) with minor modifications. To obtain the supernatant for GB analysis, the procedures were the same as described as for Cho content except that the supernatants diluted 1:1 with 2 N H_2_SO_4_. A calibration curve with reference standards of GB (5–500 μg ml^-1^) was determined.

### Determination of thiobarbituric acid-reactive substances (TBARS)

The level of lipid peroxidation was determined by thiobarbituric acid-reactive substances (TBARS) according to Madhava Rao and Sresty (2000). TBARS was calculated from the absorbance at 532 nm and measurements were corrected for nonspecific turbidity by subtracting the absorbance at 600 nm. The concentration of TBARS was calculated using an extinction coefficient of 155 mM^-1^ cm^-1^.

### Statistical analysis

The experiments were repeated twice independently, and each data point was the mean of six replicates. All data obtained were subjected to a one-way analysis of variance (one way ANOVA, SPSS Statistics 20). Tukey’s post-test was used to compare the treatment groups. Comparisons with *p* < 0.05 were considered significantly different. In all the figures, the error bars represent standard errors of the means.

## Results

**Figure 1:**
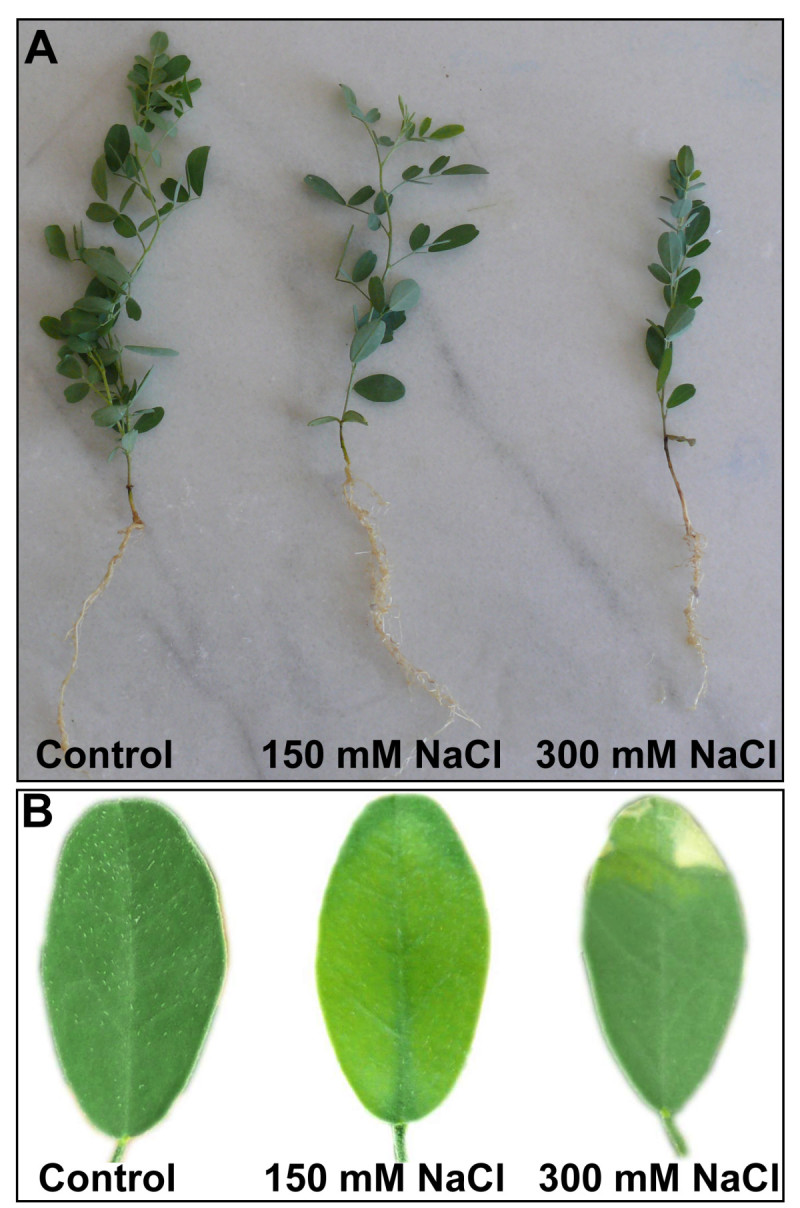
**Effects on length of seedlings (A) and leaf burns (B) in** ***S. kotschyana*** **exposed to 0 (Control), 150 mM and 300 mM NaCl for 14 d.**

Except for 7 d of 150 mM-treated plants, at which growth did not change significantly, salinity caused a decrease in the shoot and root length of *S. kotschyana* when compared with the control group. 300 mM NaCl had a significant negative effect on the growth of *S. kotschyana,* especially at 14 d (Table 1). As evidenced by phenotypic change of *S. kotschyana,* decreased length of seedlings (Figure 1A) and visible symptoms of leaf burns (Figure 1B) was more acute at 300 mM than at 150 mM NaCl. In addition, while after 14 d of stress, shoot length decreased by 25% in 300 mM NaCl-treated plants, the reduction in root length was 29% at the same day of stress exposure. As well as root and shoot length, at 14 d, 150 and 300 mM NaCl significantly decreased FW by 18% and 41% (root) and 23% and 41% (shoot), respectively. On the other hand, root/shoot ratio of dried plants (DW) increased in 150 mM-treated plants but decreased at 300 mM at 7 d as compared to the control (Table 2).

**Table 1:** **Effects on growth [length, fresh weight (FW), dry weight (DW)] and chlorophyll fluorescence (F**_**v**_ **/F**_**m**_ **) in** ***S. kotschyana*** **shoots and roots exposed to 0 (Control), 150 mM and 300 mM NaCl for 7 and 14 d**

Groups	Shoot				Root		
	Length (cm)	FW (g)	DW (g)	F_v_/F_m_	Length (cm)	FW (g)	DW (g)
**0 mM (7d)**	16.3 ± 0.7^a^	0.61 ± 0.05^a^	0.065 ± 0.004^a^	0.833 ± 0.01^a^	9.39 ± 0.73^a^	0.13 ± 0.06^a^	0.008 ± 0.001^a^
**150 mM (7d)**	16.0 ± 0.8^a^	0.55 ± 0.01^a^	0.061 ± 0.012^a^	0.841 ± 0.01^a^	9.10 ± 0.42^a^	0.12 ± 0.08^a^	0.008 ± 0.001^a^
**300 mM (7d)**	14.3 ± 0.6^b^	0.43 ± 0.01^b^	0.059 ± 0.002^a^	0.847 ± 0.01^a^	7.43 ± 0.31^b^	0.09 ± 0.01^b^	0.007 ± 0.001^a^
**0 mM (14d)**	20.4 ± 0.77^a^	0.69 ± 0.04^a^	0.09 ± 0.001^a^	0.843 ± 0.01^a^	12.4 ± 0.83^a^	0.17 ± 0.07^a^	0.011 ± 0.0020^a^
**150 mM (14d)**	19.0 ± 1.35^a^	0.53 ± 0.02^b^	0.06 ± 0.005^b^	0.844 ± 0.01^a^	9.8 ± 0.63^b^	0.14 ± 0.01^b^	0.010 ± 0.0002^a^
**300 mM (14d)**	15.2 ± 1.15^b^	0.41 ± 0.03^c^	0.05 ± 0.008^c^	0.781 ± 0.01^b^	8.8 ± 0.57^b^	0.10 ± 0.01^b^	0.006 ± 0.0001^b^

Letters a, b and c indicate a statistically significant difference between treated plants and untreated plants. The different letters are significantly different (*P* <0.05).

**Table 2:** **Effects on root/shoot ratio of length, fresh weight (FW) and dry weight (DW) in** ***S. kotschyana*** **shoots and roots exposed to 0 (Control), 150 mM and 300 mM NaCl for 7 and 14 d (days)**

	7 Days			14 Days		
	0 mM	150 mM	300 mM	0 mM	150 mM	300 mM
**Root length/shoot length**	0.576 ± 0.04^a^	0.568 ± 0.02^a^	0.519 ± 0.01^b^	0.607 ± 0.02^a^	0.515 ± 0.03^b^	0.528 ± 0.002^c^
**Root FW/shoot FW**	0.213 ± 0.01^a^	0.218 ± 0.01^a^	0.209 ± 0.02^b^	0.246 ± 0.02^a^	0.264 ± 0.03^b^	0.243 ± 0.003^a^
**Root DW/shoot DW**	0.123 ± 0.01^a^	0.131 ± 0.02^c^	0.118 ± 0.02^b^	0.122 ± 0.02^a^	0.167 ± 0.03^c^	0.12 ± 0.002^a^

Letters a, b and c indicate a statistically significant difference between treated plants and untreated plants. The different letters are significantly different (*P* <0.05).

**Figure 2:**
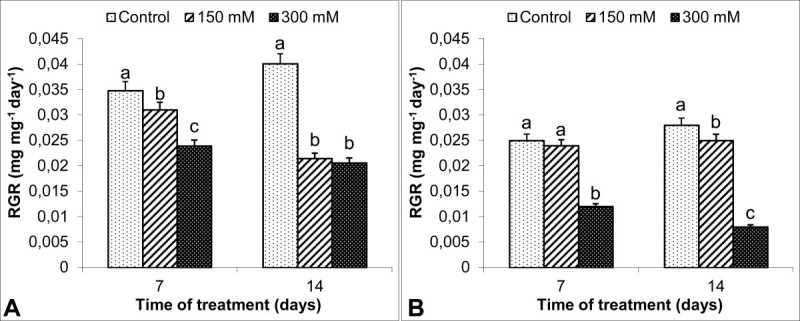
**Effects on relative growth rate (RGR, mg mg**^**-1**^**d**^**-1**^**) in** ***S. kotschyana*** **shoots (A) and roots (B) exposed to 0 (Control), 150 mM and 300 mM NaCl for 7 and 14 d are shown.** Vertical bars indicate ± SE and values sharing a common letter are not significantly different at *P >* 0.05.

All the salt treatments caused a significant reduction in RGR in comparison to the control group (Figure 2). Further decline (49%) in RGR of shoots was observed in *S. kotschyana* treated with 300 mM NaCl for 14 d. Similarly, the reduction in RGR of roots was more pronounced at 300 mM NaCl especially for 14 d, at which it attained 71% of control.

**Figure 3:**
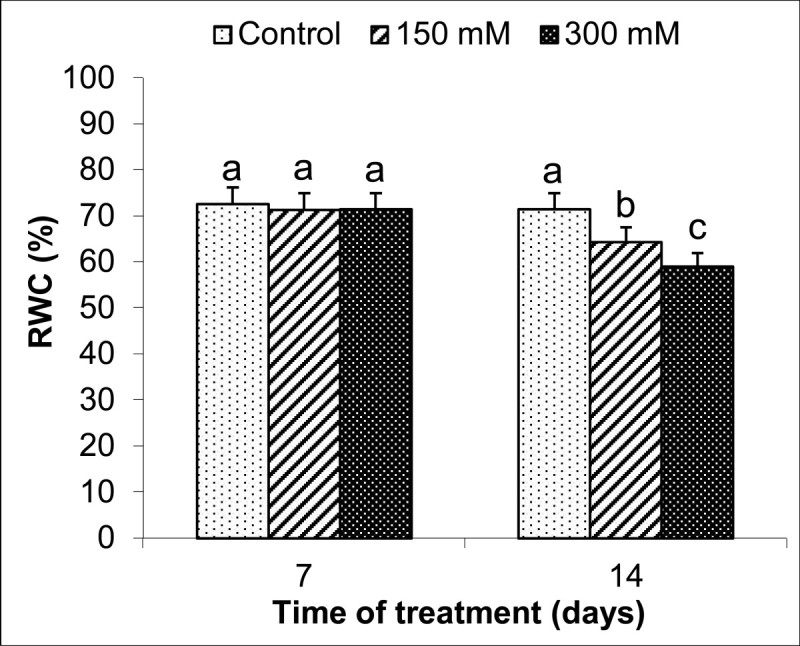
**Effects on relative water content (RWC,%) in** ***S. kotschyana*** **leaves exposed to 0 (Control), 150 mM and 300 mM NaCl for 7 and 14 d.** Vertical bars indicate ± SE and values sharing a common letter are not significantly different at *P >* 0.05.

As is evident from Figure 3, RWC was close to the control group after 7 d of stress, but it decreased at 14 d. RWC was 10% and 17% lower than in the control group at 150 and 300 mM NaCl at 14 d, respectively.

As is clear from Table 1, F_v_/F_m_ did not change at 150 mM NaCl throughout the experiment and PS-II efficiency was preserved. However, at 300 mM NaCl, F_v_/F_m_ decreased after 14 d of stress (7%).

**Figure 4:**
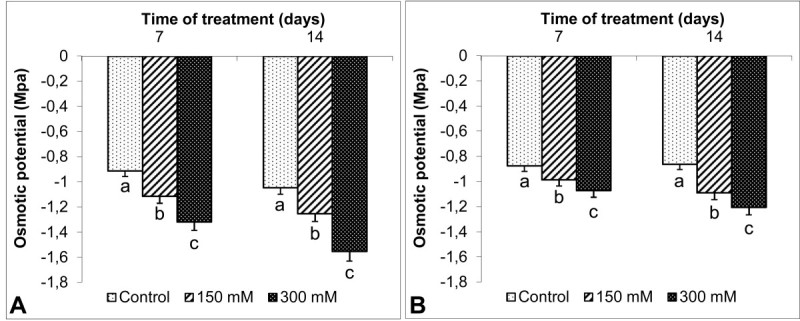
**Effects on osmotic potential (Ψ**_**Π**_**, MPa) in** ***S. kotschyana*** **leaves (A) and roots (B) exposed to 0 (Control), 150 mM and 300 mM NaCl for 7 and 14 d are shown.** Vertical bars indicate ± SE and values sharing a common letter are not significantly different at *P >* 0.05.

Ψ_Π_ in leaves and roots showed the same changes at both sampling days and salt concentrations (Figure 4). The Ψ_Π_ in leaves decreased after 7 d exposure to 150 and 300 mM. Ψ_Π_ in leaves dropped from -1.1 (control levels) to -1.6 MPa after 14 d of 300 mM NaCl. Similarly, 300 mM NaCl caused more reduction in root Ψ_Π_ (-1.2 MPa) than that of control (-0.9 MPa) at 14d. However, the reductions were more pronounced in leaves than in roots. For example, 300 mM NaCl yielded a larger Ψ_Π_ reduction (46%) in *S. kotschyana* leaves at 14 d when compared to the Ψ_Π_ of roots (33%).

**Figure 5:**
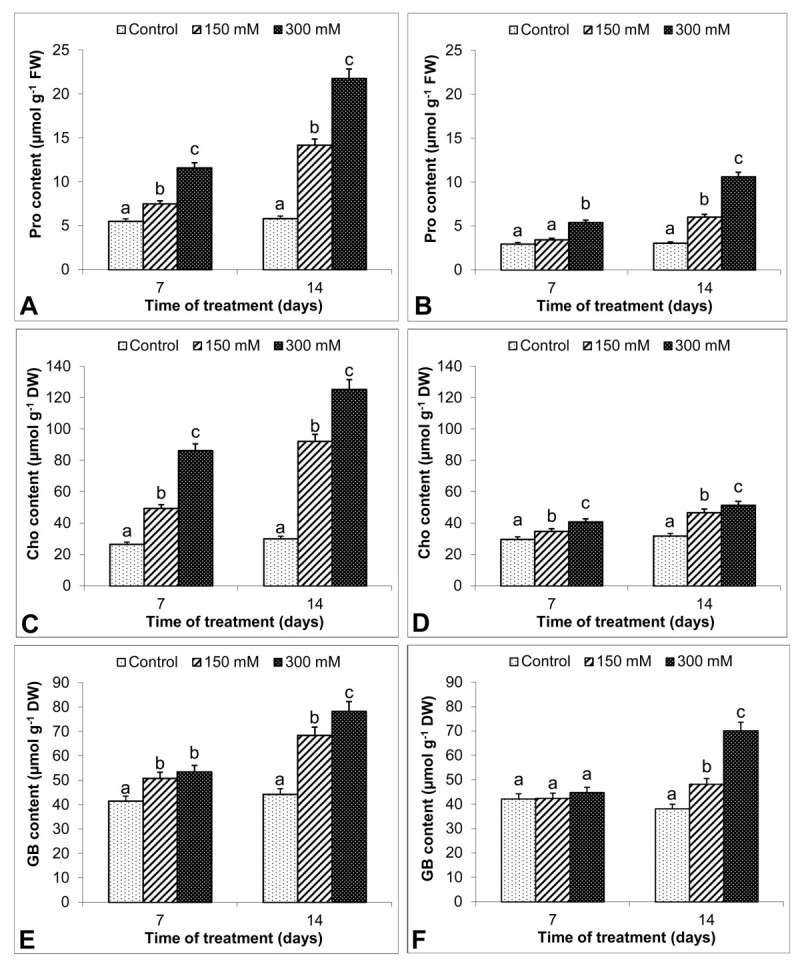
**Effects on proline (Pro, μmol g**^**-1**^**FW), choline (Cho, μmol g**^**-1**^**DW) and glycine betaine (GB, μmol g**^**-1**^**DW) in** ***S. kotschyana*** **leaves (A, C, E) and roots (B, D, F) exposed to 0 (Control), 150 mM and 300 mM NaCl for 7 and 14 d are shown.** Vertical bars indicate ± SE and values sharing a common letter are not significantly different at *P >* 0.05.

Pro, Cho and GB contents in leaves and roots increased with salinity during the experimental period (Figure 5). Even greater contents of the osmo-protectants were observed in 300 mM-treated leaves at 14 d (Figure 5A, C, E). Under 150 mM, Pro in roots was close to control level at 7d (Figure 5B). On the other hand, except this group, 150 and 300 mM NaCl increased Pro relative to control group, more so after 14 d exposure to 300 mM (274% and 247%, leaves and roots, respectively). Cho accumulation showed a significant increase in response to salinity in both leaves and roots. At 150 mM NaCl, Cho increased 1.9 and 3 fold in leaves after 7 and 14 d of the beginning of the salinity treatments as compared with the control, respectively (Figure 5C). Also, Cho content increased in roots of stress-treated *S. kotschyana*, but this induction was as high as that in leaves (Figure 5D). For example, when NaCl stress became more severe, the increase in leaves (315%) was much greater than in root (62%) at 14 d. On the other hand, GB in leaves markedly increased in response to 150 and 300 mM NaCl and accumulated to significantly higher levels at the higher salt concentration (Figure 5E). GB levels in roots did not change with salinity at 7 d (Figure 5F). In contrast, GB concentration showed a dramatic increase in response to 300 mM. The highest induction in roots (84%) was obtained at 14 d.

**Figure 6:**
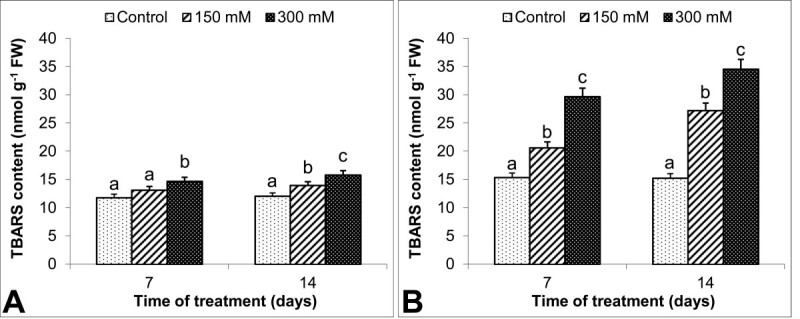
**Effects on TBARS content (nmol g**^**-1**^**FW) in** ***S. kotschyana*** **leaves (A) and roots (B) exposed to 0 (Control), 150 mM and 300 mM NaCl for 7 and 14 d are shown.** Vertical bars indicate ± SE and values sharing a common letter are not significantly different at *P* > 0.05.

Except in leaves of 150 mM-treated plants at 7 d, TBARS in leaves and roots increased with salinity as compared to control group (Figure 6A, B). Moreover, 300 mM NaCl induced the maximum rate of lipid peroxidation in membranes and caused an increase in damage by 31% in leaves at 14 d when compared to the control. On the other hand, the highest increase in TBARS in roots was 126% at the same days of stress.

## Discussion

Growth of *S. kotschyana* was significantly reduced with NaCl treatments in a dose-dependent manner, which is in agreement with different studies (Athar et al., 2009; Noreen et al., 2010). This change might be due to toxic effects of Na^+^ and Cl^¯^ and unbalancing of nutrient uptake. A previous study carried out with cotton (Meloni et al., 2003) also showed that shoot growth was more inhibited by NaCl than that of root. In our experiment, while the reduction percentages of root lengths were parallel to the results of previous studies, changes in FW and DW were in conflict. On the other hand, increased root/shoot ratio appears to be an adaptation to salinity, resulting in a more efficient water and nutrient uptake under stress (Gorham et al., 1985), supporting our findings, especially in 150 mM NaCl-treated plants.

Information on the relationship between salt tolerance and RWC, RGR or Ψ_Π_ would be useful to use these parameters as selection criteria in water regulations. After 14 d of stress in *S. kotschyana*, the decrease in RWC is in confirmation of results reported by Sairam et al. (2002). The decrease in RWC resulted a loss of turgor and then limited water availability for cell extension processes (Katerji et al., 2003). In the present study, the inhibition in RGR was stronger in 300 mM NaCl-treated roots of *S. kotschyana*, demonstrating results of FW and DW. This result was well in agreement with those of Kim et al. (2004) and Lacerda et al. (2005). 300 mM might inhibit the growth rate of *S. kotschyana* due to the osmotic and ionic effects of salinity in line with the findings of Sun et al. (2009). This allows soil water uptake and sustains the turgor potential. In *S. kotschyana* exposed to salinity, the increase in osmolytes was compatible with the reduction in Ψ_Π_.

F_v_/F_m_ is used as chlorophyll fluorescence parameter, closely correlated to photochemical efficiency of PSII and is a sensitive indicator of photosynthetic performance in plants (Kalaji et al., 2010). This parameter is thought to play a vital role in the process of photoinhibition when plants are exposed to drought and salt stresses (Maxwell and Johnson, 2000). F_v_/F_m_ is close to 0.83 for most plants grown without stress (Bjorkman and Demmig 1987). Values lower than 0.83 suggest that plants are growing under stress and that PSII reaction centers are damaged which, in turn, is connected with reduced effectiveness of electron transport (Basu et al., 1998) when plants are grown under salinity. Similar to this, F_v_/F_m_ showed a significantly decrease after 14 d exposure to 300 mM NaCl. However, 150 mM NaCl did not change the F_v_/F_m_ of *S. kotschyana*. It is reported by Sakamoto and Murata (2002) that the protection of PSII complexes from oxidative damage might be connected with increased GB content under salt stress.

Pro maintains NAD^+^/NADH ratios necessary for respiratory and photosynthetic processes, enhances photosystem II-mediated photochemical activity in thylakoid membranes and prevents the photoinhibitory loss of photochemical activity under stress conditions (Kavi Kishor et al., 2005). In our experiment, a dramatic increase in Pro content in the plants exposed to the highest NaCl concentration could contribute to the lack of notable change in photosynthetic efficiency. Supporting findings come from other plants such as sugar beet (Ghoulam et al., 2002) where salt stress results in extensive Pro accumulation. Also, it has been reported in salt treated *Anacardium occidentale* that Pro accumulation is higher in leaves than roots (Alvarez-Pizarro et al., 2009) which is in accordance to the results obtained in this experiment. Recent experimental evidence has demonstrated that plant species able to synthesize GB may also accumulate other organic compatible solutes, such as Pro (Martinez et al., 2005). GB has been shown *in vitro* (i) to stabilize membranes of the oxygen evolving photosystem II complex and (ii) to accumulate in the cytoplasm to balance the accumulation of solutes and ions in the vacuole during osmotic adjustment (Papageorgiou and Murata, 1995). There was a significant positive correlation among Pro, GB contents and the extent of tolerance to oxidative in *S. kotschyana*. However, it seems to be that Pro had more contribution than GB regarding osmotic adjustment. Another contributor to osmotic adjustment in salt stressed*-S. kotschyana* was Cho. Cho is synthesized from GB in the chloroplast. Like Pro and GB, Cho increased with salinity treatments in *S. kotschyana* as suggested by Varshney et al. (1988). Therefore, the induced contents of these osmo-protectants were compatible with each other under salinity. Also, due to the increase in Pro, GB and Cho contents in leaves of 150 mM-treated plants, accumulation of these osmo-protectants might be sufficient for protection of the photosynthetic apparatus and water status against oxidative damage.

Increases in TBARS have been reported in salt stress-treated plants (Sudhakar et al., 2001; Sairam et al., 2002). This increase is related to the amount of stress and is well correlated with lipid membrane damage. Roots of *S. kotschyana* seemed to be more affected by the NaCl-induced oxidative stress, as evidenced by a considerable increase in TBARS, as well as by the further decrease in RGR. Additionally, roots had a lower induction in osmo-protectant accumulation than did leaves.

## Conclusions

This is the first report to provide insights into the mechanisms of stress tolerance and to compare water relations, osmotic adjustment and photosynthetic alterations in response to salinity of *S. kotschyana*. The results clearly showed that roots would appear to be the most vulnerable part of this plant, as they were directly exposed to the salt. Thus, in summary, the degree of osmotic adjustment in leaves was greater than roots with the increase in NaCl concentration. Any increase in osmotic adjustment under saline conditions might likely be a result of increase in the ions in the vacuole. Our results support the idea that leaves and roots of *S. kotschyana* were well-adapted to saline environments, as suggested by maintenance of a high root/shoot ratio, lower increase in TBARS accumulation, lower decline in RGR, plant growth parameters and Ψ_Π_ especially on 7 d. Therefore, *S. kotschyana* could withstand both short-term salinity and low salt concentration. Therefore, the amounts of osmo-protectants in *S. kotschyana*’s response to salinity acclimation or adaptation were sufficient to for tolerance of low salt concentration (150 mM). In contrast, in the plants exposed to 300 mM NaCl, despite the increase in osmolyte accumulation, was not able to maintain the ability to prevent water loss and became oxidatively damaged as demonstrated by a further decrease in growth, root/shoot ratio, RGR, RWC and a decline in antioxidant enzymes activity.

## Authors’ original submitted files for images

Below are the links to the authors’ original submitted files for images. Authors’ original file for figure 1 Authors’ original file for figure 2 Authors’ original file for figure 3 Authors’ original file for figure 4 Authors’ original file for figure 5 Authors’ original file for figure 6

## Abbreviations

Cho: Choline
GB: Glycine betaine
Pro: Proline
RGR: Relative growth rate
RWC: Relative water content
TBARS: Thiobarbituric acid-reactive substances.
