# Supplementary figures and images for: Variations in osmotic adjustment and water relations of Sphaerophysa kotschyana: Glycine betaine, proline and choline accumulation in response to salinity
Source: Bot Stud. 2014 Jan 17;55:6. doi: 10.1186/1999-3110-55-6 (PMC5432748; doi:10.1186/1999-3110-55-6)

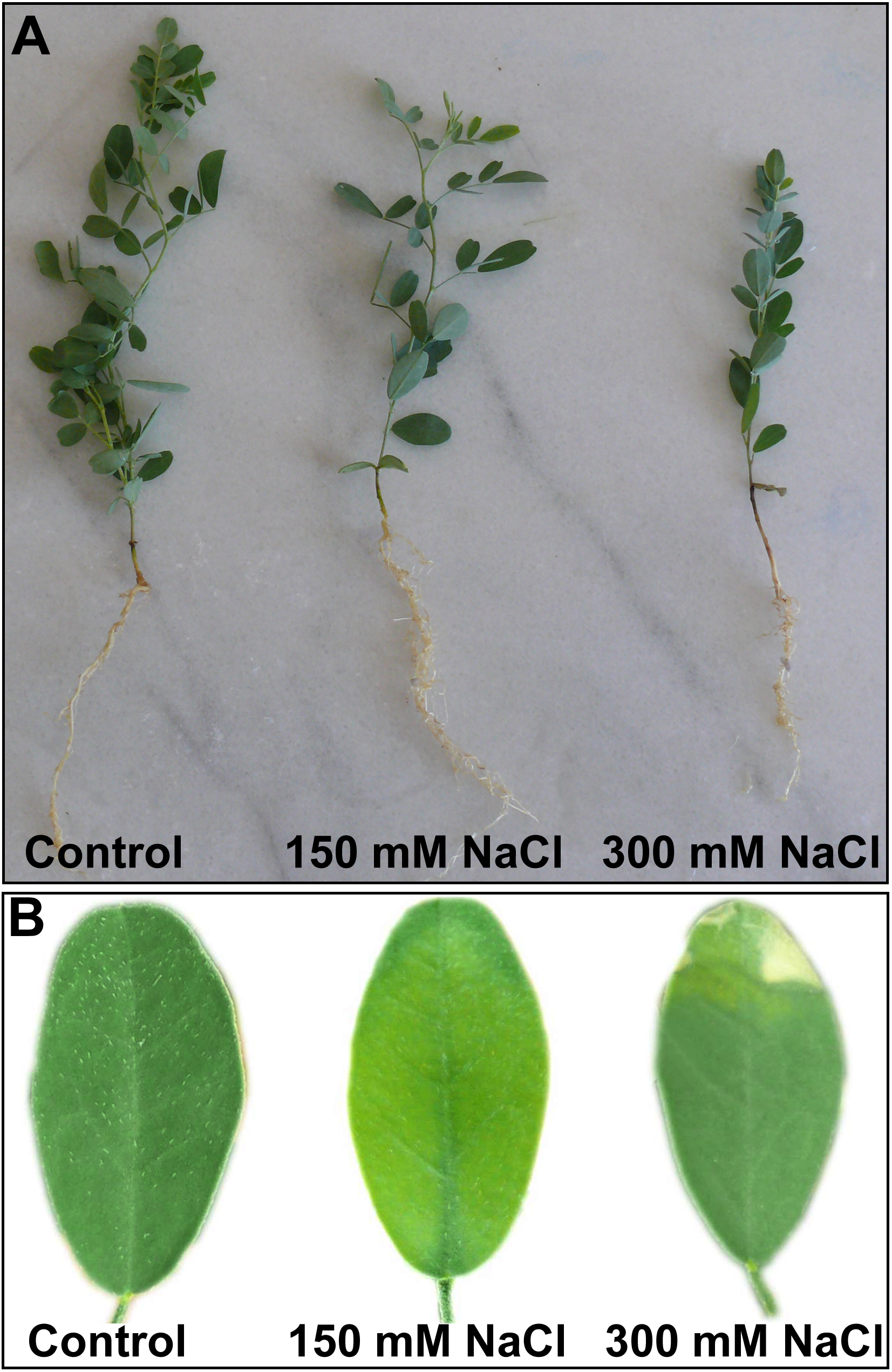

Supplement: Supplementary file 1 — Authors’ original file for figure 1 [file 40529_2013_56_MOESM1_ESM.tif]

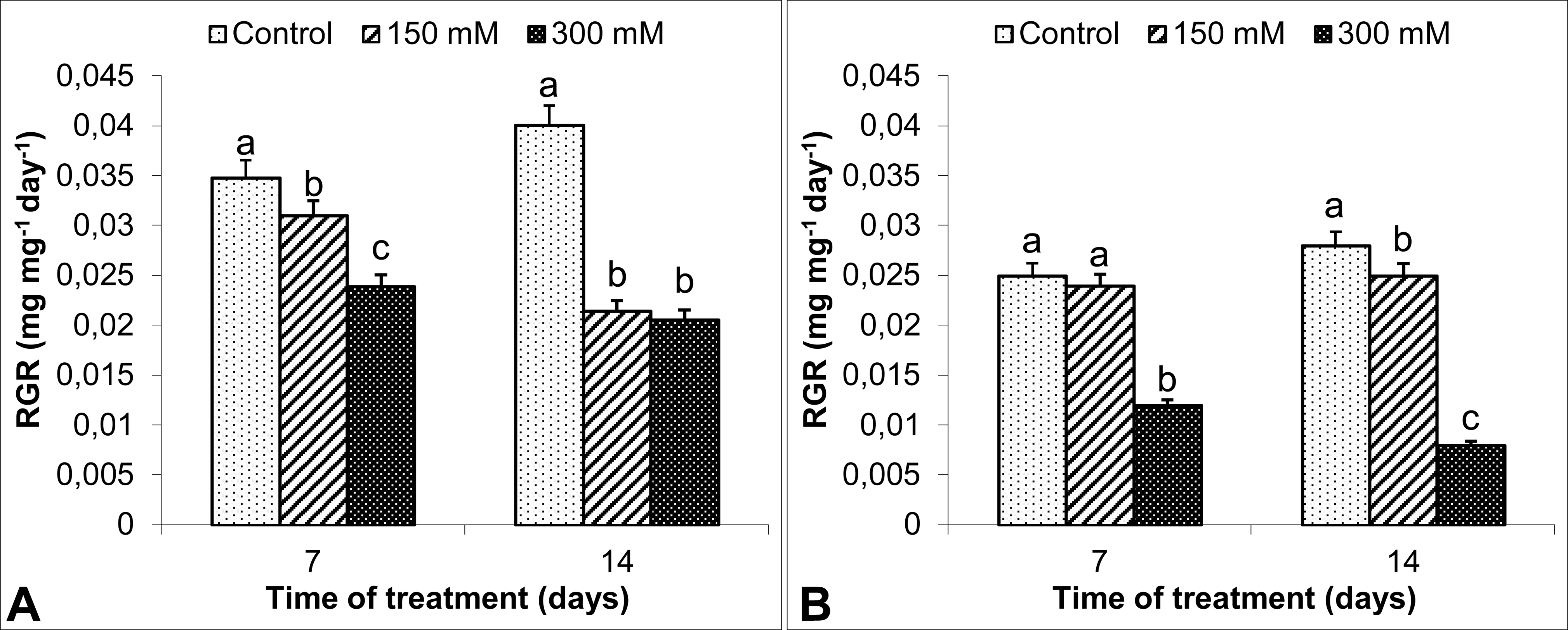

Supplement: Supplementary file 2 — Authors’ original file for figure 2 [file 40529_2013_56_MOESM2_ESM.tiff]

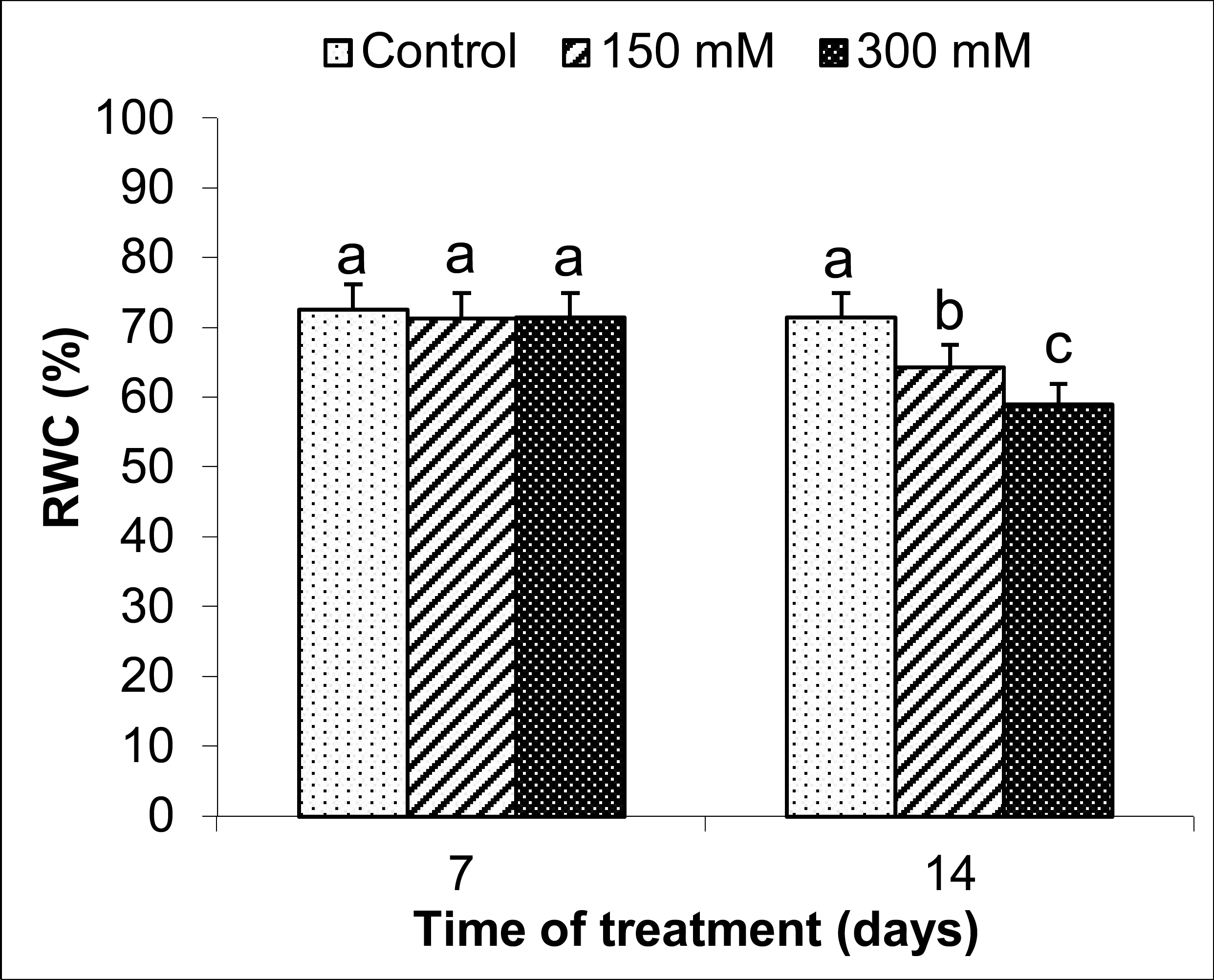

Supplement: Supplementary file 3 — Authors’ original file for figure 3 [file 40529_2013_56_MOESM3_ESM.tiff]

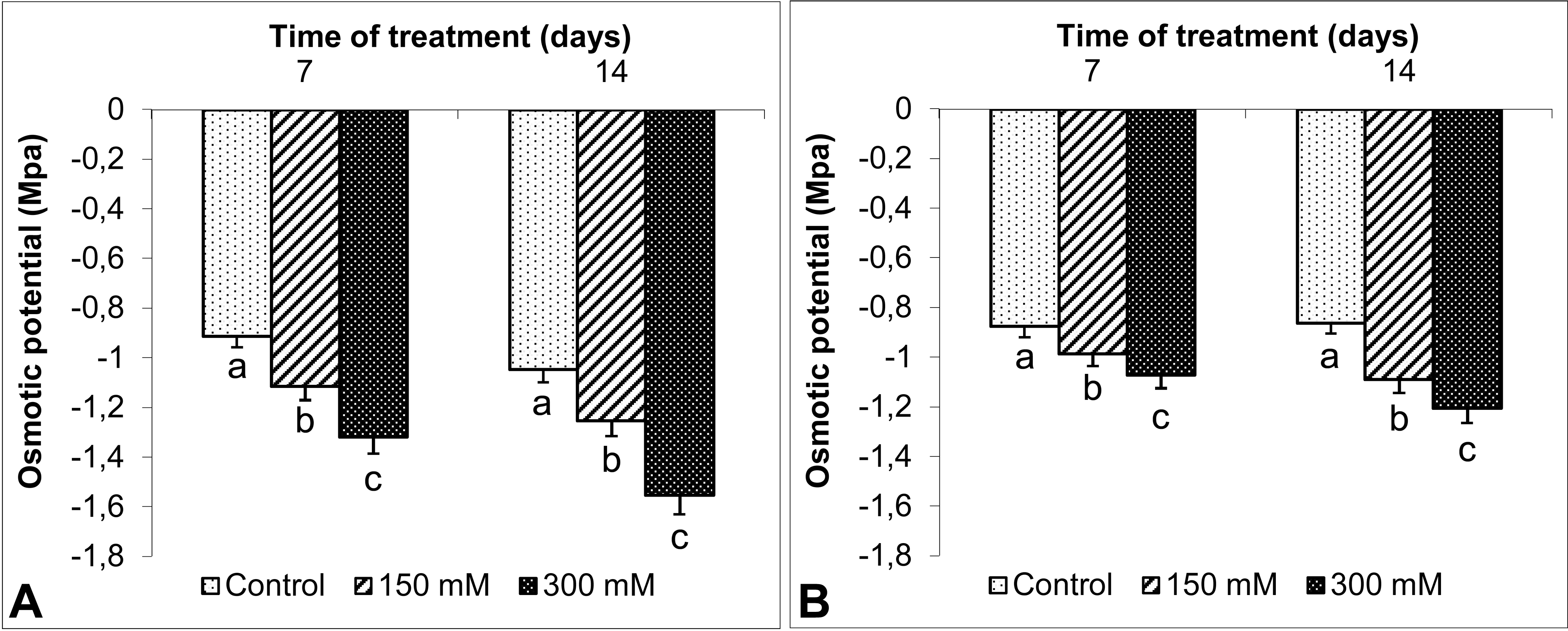

Supplement: Supplementary file 4 — Authors’ original file for figure 4 [file 40529_2013_56_MOESM4_ESM.tif]

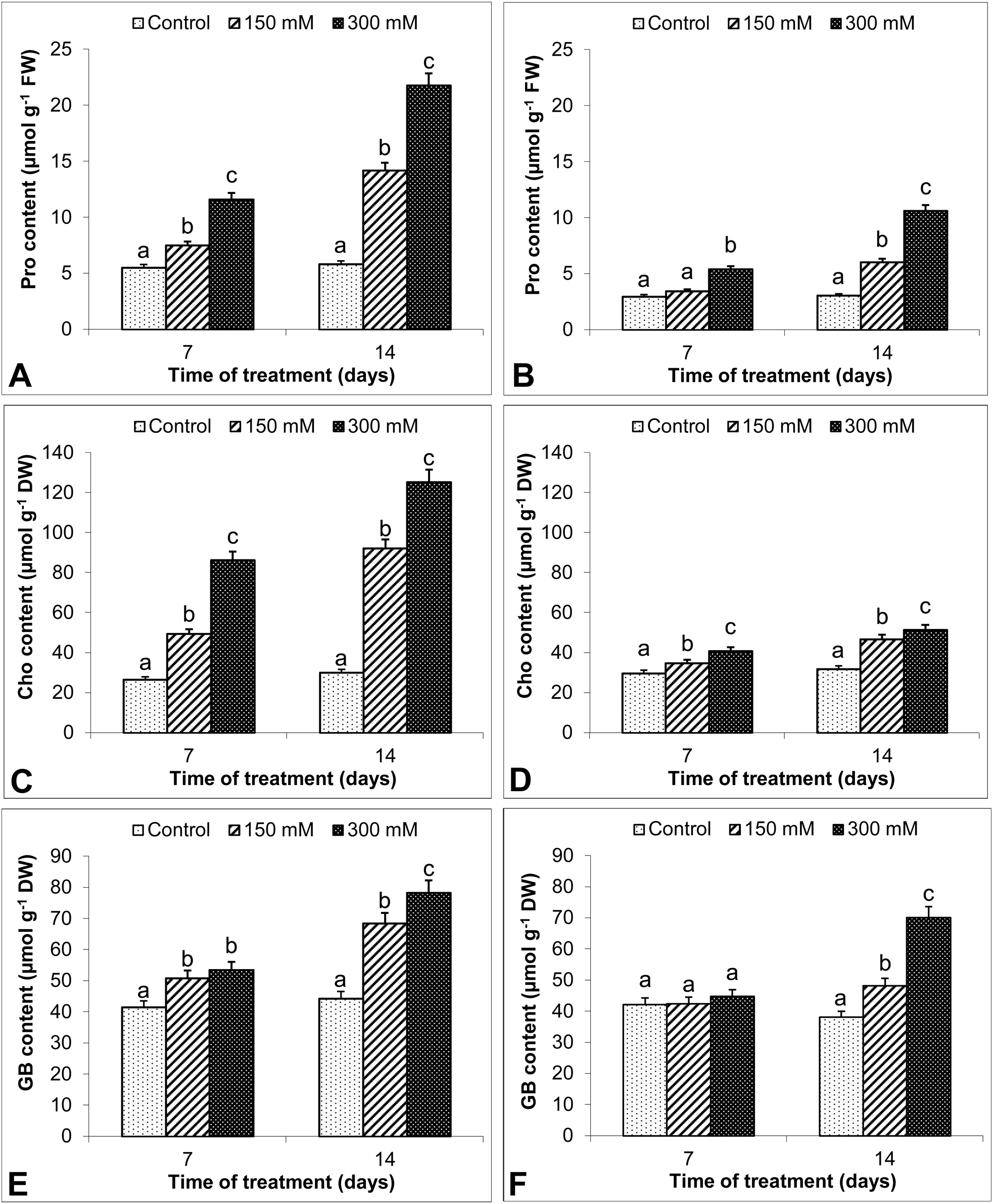

Supplement: Supplementary file 5 — Authors’ original file for figure 5 [file 40529_2013_56_MOESM5_ESM.tif]

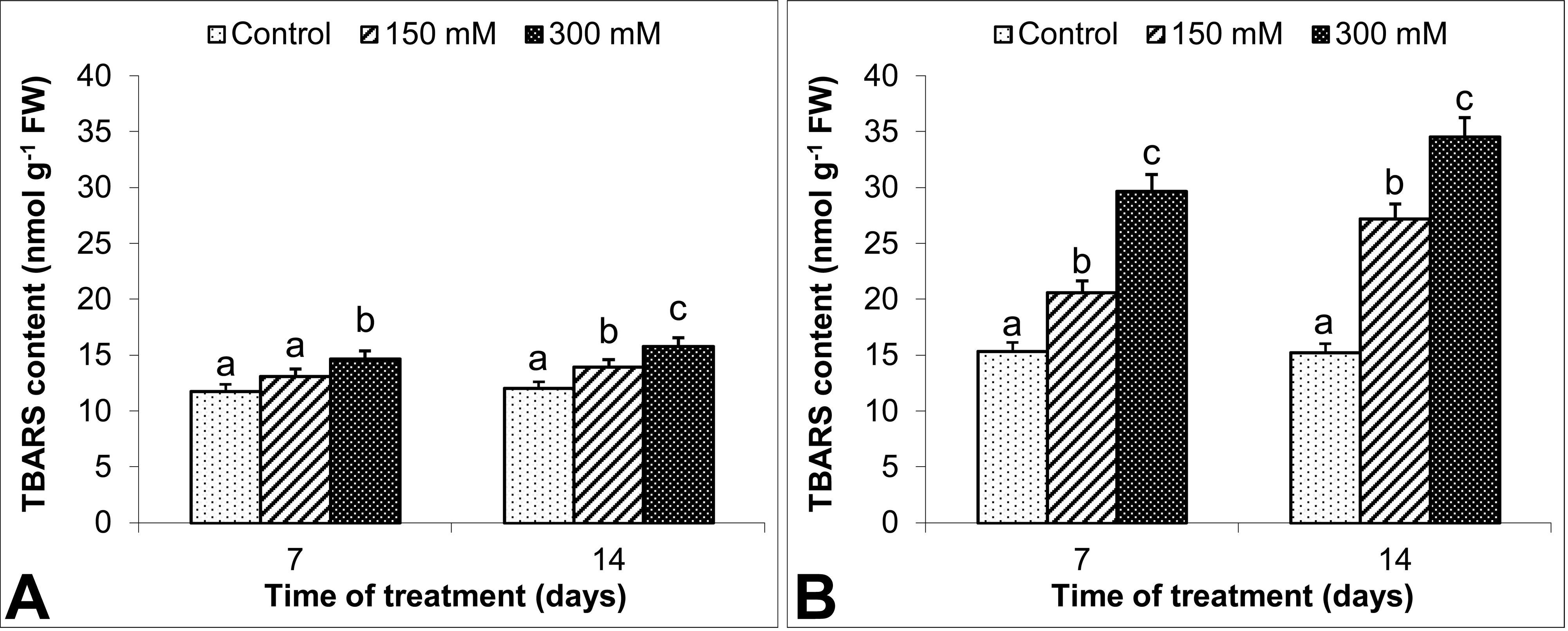

Supplement: Supplementary file 6 — Authors’ original file for figure 6 [file 40529_2013_56_MOESM6_ESM.tif]
